# Ultrasound evaluation of diaphragmatic dysfunction

**DOI:** 10.1590/0100-3984.2016.0072

**Published:** 2017

**Authors:** Rachel Zeitoune, Ana Célia Baptista Koifman, Marina Shu Fong, Roberto Mogami

**Affiliations:** 1 Hospital Universitário Pedro Ernesto (HUPE), Rio de Janeiro, RJ, Brazil; 2 Hospital Universitário Gaffrée & Guinle (HUGG), Rio de Janeiro, RJ, Brazil

*Dear Editor*,

A 49-year-old male patient presented with a complaint of dyspnea when swimming, which he did regularly. The following were performed: chest X-ray, which showed elevation of the right hemidiaphragm; respiratory function tests, which revealed mild restrictive lung disease; and ultrasound of the diaphragm, which demonstrated a significant reduction in the mobility of the right hemidiaphragm, although not to the point of paralysis.

Ultrasound of the diaphragm has been used mainly in patients in intensive care. In such patients, assessment of the diaphragm by ultrasound can be used in order to predict successful weaning from mechanical ventilation^(1)^, to inform decisions regarding adjustments in mechanical ventilation parameters, and to investigate postoperative weakness/diaphragmatic paralysis^(2)^, as well as to identify diaphragmatic atrophy after prolonged mechanical ventilation^(3)^. In the present report, two radiologists evaluated the thickness and mobility of the diaphragm, using B-mode and M-mode ultrasound, respectively. The evaluations were made by consensus.

In the B-mode evaluation, the hemidiaphragms were accessed via the intercostal spaces. With the patient in the supine position, a linear multifrequency (7-18 MHz) transducer was positioned in the longitudinal plane on the anterior axillary line, between the 7th and 8th or 8th and 9th intercostal spaces^(4)^. Assessments were made at the zone of apposition, where the diaphragm abuts the lower rib cage^(5)^. The normal diaphragm was visualized between two echogenic lines^(1)^: that of the parietal pleura and that of the peritoneal membrane. Three measurements of muscle thickness were performed during maximal inspiration and expiration maneuvers, and the unweighted mean was calculated for each maneuver. During inspiration, there was contraction and shortening of the fibers of the normal diaphragm, with increased muscle volume and consequent thickness. We calculated the diaphragm thickening fraction (DTF, defined as *inspiratory thickness − expiratory thickness* / *expiratory thickness* × 100)%, which quantifies the degree of muscle thickening from functional residual capacity to total lung capacity, using the mean of the measurements^(5)^.

In the M-mode evaluation, the right hemidiaphragm was accessed via the anterior subcostal route, obliquely between the hemiclavicular and anterior axillary lines, and the left hemidiaphragm was accessed via the intercostal route, on the middle axillary line. In either case, the patient was placed in the supine position and a convex 2-5 MHz transducer was used^(4)^. Curves of the diaphragm kinetics were acquired under three respiratory conditions^(2)^: quiet breathing, deep breathing, and sniffing. In each condition, we obtained three waves and their respective amplitudes, calculating the unweighted mean of the measurements.

**Figure 1:**
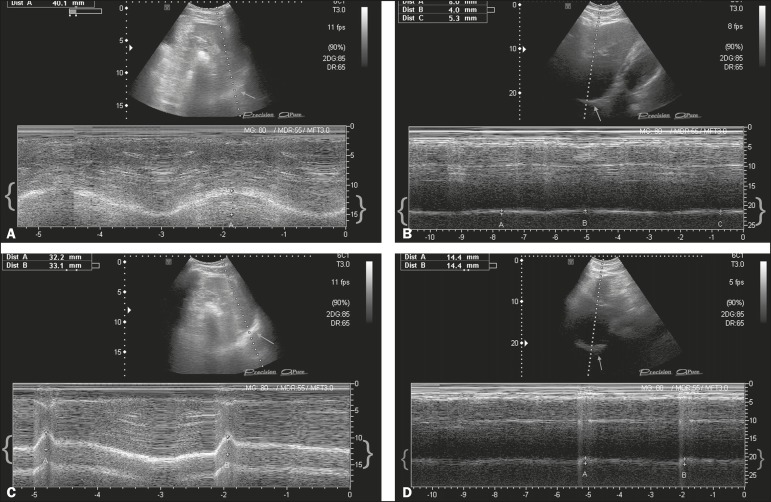
**A:** M-mode ultrasound during quiet breathing. Left hemidiaphragm. The curve between braces represents the normal trajectory of diaphragm mobility during quiet breathing. The arrow shows the left hemidiaphragm, accessed via the anterior subcostal route, in the oblique plane with a spleen window, in B mode. **B:** M-mode ultrasound during quiet breathing. Right hemidiaphragm. The curve between braces shows that there was a major reduction in the mobility of the right hemidiaphragm. The arrow shows the right hemidiaphragm, accessed via the subcostal route, with a liver window, in B mode. **C:** M-mode ultrasound during sniffing. Left hemidiaphragm. The curve between braces represents the normal trajectory of diaphragm mobility during sniffing. The arrow shows the left hemidiaphragm, accessed via the anterior subcostal route, in the oblique plane with a spleen window, in B mode. **D:** M-mode ultrasound during sniffing. Right hemidiaphragm. The curve between braces shows that there was a major reduction in the mobility of the right hemidiaphragm during sniffing. The arrow shows the right hemidiaphragm, accessed via the subcostal route, with a liver window, in B mode.

The maximum inspiratory and expiratory thickness of the diaphragm was 0.29 cm and 0.22 cm, respectively, for the right hemidiaphragm, compared with 0.35 cm and 0.20 cm, respectively, for the left hemidiaphragm. The DTF was 31% and 73% for the right and left hemidiaphragms, respectively. In the quiet breathing, deep breathing, and sniffing conditions, the mobility of the diaphragm was 0.57 cm, 2.24 cm, and 1.24 cm, respectively, for the right hemidiaphragm, compared with 4.53 cm and 3.44 cm for the left hemidiaphragm in the quiet breathing and sniffing conditions, respectively (Figure 1). It was not possible to determine the mobility of the left hemidiaphragm during deep breathing, probably due to the small size of the (spleen) window.

In the case reported here, the mobility of the right hemidiaphragm was significantly reduced. However, we did not identify diaphragmatic paralysis, the diagnostic criteria for which are a DTF below 20% in B-mode^(5)^ and paradoxical breathing, characterized by a curve below the baseline in M-mode^(6)^. At this writing, the patient is being monitored and is under conservative treatment, showing gradual clinical improvement.
